# EARLY-LIFE MILITARY EXPOSURES AND HEALTH AMONG OLDER VETERANS: THE BUFFERING EFFECT OF PSYCHOLOGICAL RESILIENCE

**DOI:** 10.1093/geroni/igz038.1167

**Published:** 2019-11-08

**Authors:** Miles G Taylor, Stephanie Ureña, Dawn Carr, Stella N Min

**Affiliations:** 1 Florida State University, Tallahassee, Florida, United States; 2 Department of Sociology, Florida State University, Tallahassee, Florida, United States

## Abstract

Objectives Drawing on the life course framework and theoretical concept of resilience, we examine the impact of early-life service-related exposures (SREs) on later-life functional impairment trajectories among older U.S. male veterans. We conceptualize resilience as a psychological resource potentially moderating the lasting negative consequences of traumatic military exposures. Method Using the 2013 Veterans Mail Survey linked to the Health and Retirement Study 2006–2014 Leave Behind Questionnaire and RAND Data File (v.N), we estimate latent growth curve models of functional impairment trajectories. Results SRE to death has a persistent positive effect on functional limitations and activities of daily living limitations. Psychological resilience significantly moderates this association, such that veterans maintaining higher levels of resilience in the face of adverse exposures have considerably less functional impairment over time compared to their counterparts with low levels of resilience. Discussion Our findings point to the importance of psychological resilience in later life, especially within the realm of traumas occurring in early life. We discuss implications for current military training programs, stressing the importance of research considering individual resources and processes that promote adaptation in the face of adverse life events.

